# Effect of Coxsackievirus B4 Infection on the Thymus: Elucidating Its Role in the Pathogenesis of Type 1 Diabetes

**DOI:** 10.3390/microorganisms9061177

**Published:** 2021-05-29

**Authors:** Abdulaziz Alhazmi, Magloire Pandoua Nekoua, Hélène Michaux, Famara Sane, Aymen Halouani, Ilka Engelmann, Enagnon Kazali Alidjinou, Henri Martens, Hela Jaidane, Vincent Geenen, Didier Hober

**Affiliations:** 1 Laboratoire de Virologie ULR3610, Université de Lille, CHU Lille, F-59000 Lille, France; abalhazmi@jazanu.edu.sa (A.A.); magloire-pandoua.nekoua@univ-lille.fr (M.P.N.); famara.sane@chru-lille.fr (F.S.); ilka.engelmann@chru-lille.fr (I.E.); enagnonkazali.alidjinou@chru-lille.fr (E.K.A.); 2 Microbiology and Parasitology Department, College of Medicine, Jazan University, Jazan 82911, Saudi Arabia; 3 GIGA-I3 Center of Immunoendocrinology, GIGA Research Institute, University of Liège, 4000 Liège, Belgium; hmichaux88@gmail.com (H.M.); hmartens@uliege.be (H.M.); vgeenen@uliege.be (V.G.); 4 Laboratoire des Maladies Transmissibles et Substances Biologiquement Actives LR99ES27, Université de Monastir, 5000 Monastir, Tunisia; halouani.aymen@yahoo.com (A.H.); jaidanehela@yahoo.fr (H.J.)

**Keywords:** viruses, enterovirus, Coxsackievirus B, thymus, self-tolerance, autoimmunity, type diabetes, fetal and perinatal life

## Abstract

The thymus gland is a primary lymphoid organ for T-cell development. Various viral infections can result in disturbance of thymic functions. Medullary thymic epithelial cells (mTECs) are important for the negative selection of self-reactive T-cells to ensure central tolerance. Insulin-like growth factor 2 (IGF2) is the dominant self-peptide of the insulin family expressed in mTECs and plays a crucial role in the intra-thymic programing of central tolerance to insulin-secreting islet β-cells. Coxsackievirus B4 (CVB4) can infect and persist in the thymus of humans and mice, thus hampering the T-cell maturation and differentiation process. The modulation of IGF2 expression and protein synthesis during a CVB4 infection has been observed in vitro and in vivo in mouse models. The effect of CVB4 infections on human and mouse fetal thymus has been studied in vitro. Moreover, following the inoculation of CVB4 in pregnant mice, the thymic function in the fetus and offspring was disturbed. A defect in the intra-thymic expression of self-peptides by mTECs may be triggered by CVB4. The effects of viral infections, especially CVB4 infection, on thymic cells and functions and their possible role in the pathogenesis of type 1 diabetes (T1D) are presented.

## 1. Introduction

Enteroviruses (EVs) are small, non-enveloped, single-stranded, positive-sense RNA viruses that belong to the *Picornaviridae* family. The Enterovirus genus comprises 15 species including 7 species involved in human diseases (Enterovirus A–D and Rhinovirus A–C). Group B coxsackieviruses encompassing six serotypes (CVB 1-6) are classified within the Enterovirus-B species [1,2]. These viruses are ubiquitous and responsible for a wide range of infections ranging from asymptomatic and mild diseases to serious illnesses. EVs have been linked to some chronic diseases such as dilated cardiomyopathy and type 1 diabetes mellitus (T1D) [3]. T1D is a multifactorial disease leading to β-cell destruction that disrupts insulin production. EV infections have been proposed as an important environmental factor with a major role in T1D pathogenesis [4]. Stewart et al. provide a link between viral infection and autoimmune diabetes by developing transgenic mice in which the insulin-producing β-cells express interferon-α (IFN-α). Local production of this antiviral cytokine by β-cells could trigger an inflammatory reaction involving the activation of natural killer cells, macrophages and T-cells, leading to hypoinsulinemic diabetes [5]. Horwitz et al. found that bystander activation could have a role in the activation of autoreactive T-cells against β-cells, owing to the initial viral infection. This infection could create an inflammatory microenvironment by stimulating the production of immune system factors such as cytokines and chemokines. In susceptible individuals, autoreactive T-cells that escaped thymic selection might become non-specifically stimulated. Eventually, those autoreactive T-cells will be activated owing to the pro-inflammatory microenvironment [6]. Molecular mimicry has also been proposed as a possible mechanism of T1D, in which a viral infection can stimulate an aggressive response of T-cells. Indeed, viruses may carry an epitope that strongly resembles certain structures of β-cells, leading to auto-reaction of T-cells against both viral and β-cell epitopes [7,8]. The thymus gland plays a major role in central tolerance through immunologic self and non-self-discrimination ability and self-specific regulatory T-cells (Treg) positive selection in perinatal life. However, T-cell selection in the thymus can be disrupted by some viral infections [9]. The persistence of EVs in pancreatic tissues has been demonstrated in human and animal models [10,11]. Moreover, it has been suggested that exposure to EVs in the earliest phase during fetal growth may lead to a defect in intra-thymic self-presentation, in turn leading to auto-destruction of β-cells by self T-cells [12].

In this review, after a presentation of the function of the thymus, the effects of viral infections and, especially, coxsackievirus B4 (CVB4) infections on thymic cells and thymic functions and the potential results of these effects in the pathogenesis of T1D are presented.

## 2. Thymic Structure and Function

### 2.1. Thymic Structure

**Figure 1:**
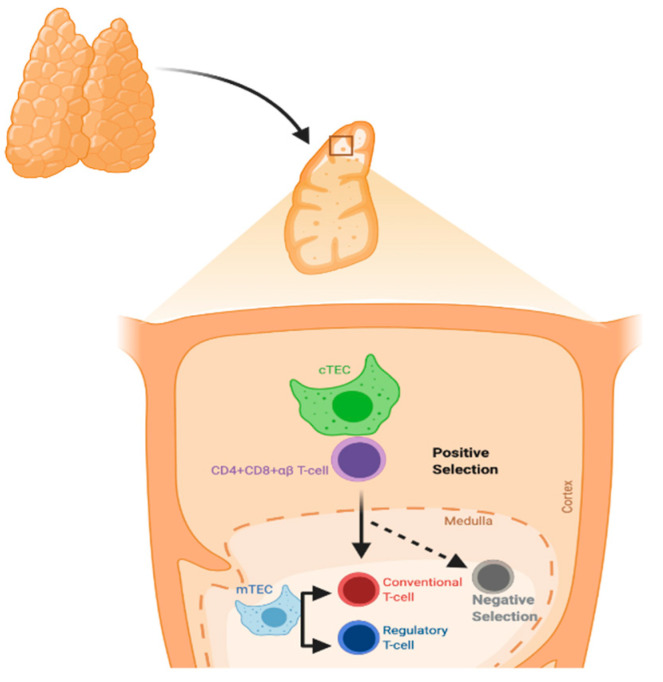
Process of T-cell selection in the thymus. Positive selection mainly occurs in cortical thymic epithelial cells (cTECs), whereas negative selection occurs in medullary TECs (mTECs).

The thymus gland is a primary lymphoid organ for T-cell development. It has two lobes divided into multiple lobules. The lobule is the structural unit of the thymus, and it comprises the cortex and medulla. The cortex is rich in immature T-lymphocytes, while the medulla has only a few mature T-lymphocytes. Thymic epithelial cells (TECs) are divided according to their localization into cortical and medullary TECs (cTECs and mTECs, respectively) and account for most thymic stromal populations (Figure 1) [13].

The major histocompatibility complex (MHC) molecules present neuroendocrine self-peptides to T-cells in the thymus. Self-reactive T-cell clones, i.e., T-cells recognizing the host molecules, are deleted and Treg cells are generated. Treg cells can inhibit self-reactive T-cells that evade thymic clonal deletion. Intra-thymic transcription is regulated by the autoimmune regulator protein (AIRE), and in particular concerns, all the members of the insulin gene family [14].

### 2.2. T-Cells Maturation and Differentiation

**Figure 2:**
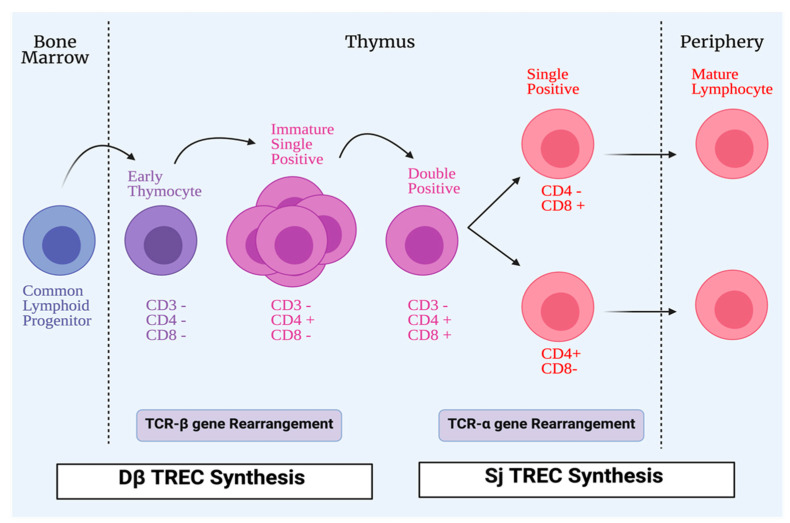
Process of T-cell maturation. All conventional T-cells start life as lymphoid progenitors, which migrate from the bone marrow to the thymus to initiate proliferation and maturation. In the thymus, early thymocytes lack the expression of CD4 and CD8 co-receptors that are involved in T-cells receptor (TCR) signaling. The TCR, which has two chains, is formed by a process of TCR-β gene rearrangement. Early thymocytes then develop CD4+CD8+ (double positive), and during that time the TCR-α chain develops. CD4+CD8+ (double positive) cells differentiate into single positive cells, either CD4+CD8- or CD4-CD8+ to be exported as mature lymphocytes from the thymus to the periphery. During TCR rearrangements, some DNA small circles are formed in T-cells during their passage in the thymus; they are called T-cell receptor excision circles (TRECs). Dβ TRECs are synthesized during TCR-β rearrangement, whereas sj TRECs are synthesized during TCR-α rearrangement.

Hematopoietic precursors within the thymus, derived from the bone marrow, can differentiate and generate αβ- or γδ- mature T-cells. The T-cell maturation process in the thymus is considered one of the important events leading to the development of a functional and effective immune system. DNA recombination is responsible for the generation of lymphocyte antigen receptors; cells with these receptors can identify and interact with a wide range of diverse molecules. T-cells are subjected to different steps of maturation in the lymphoid organs during the T-cell maturation process [15]. During this process, generation of a pool of mature and functional T-cells occurs through two different mechanisms. T-cells expressing competent receptors are selected: this process occurs in the cortex and is called positive selection. In contrast, self-reactive T-cells are deleted: this process called negative selection, occurs in the medulla and is needed for ensuring central tolerance. Owing to DNA rearrangements, immature T-cells can express different T-cell receptors (TCR). However, positively selected cells can recognize host MHC molecules [16]. Recombination-activating genes 1 and 2 (RAG1 and RAG2), in the thymic cortex, are stimulated to produce random recombination of V, D, and J segments, which leads to T-cells expressing both a pre-TCR and the co-receptors CD4 and CD8. The rearrangement of the TCR-α chain continues until a productive interaction between the TCRαβ complex and MHC complex of cTECs occurs; this is recognized as positive selection of the T-cell. The absence of a productive TCR rearrangement among thymocytes leads to apoptosis. However, the outcome of the thymocyte is determined by the interactions between the T-cells and cTECs [17]. During TCR rearrangement, some small circles of DNA are created in T-cells during their passage through the thymus; they are called T-cell receptor excision circles (TRECs) (Figure 2).

These TRECs may be reduced in some congenital diseases and infections [18]. Thymic functions can be assessed with the quantification of TRECs signal joint T-cell receptor excision circles (sjTRECs) and DβTRECs in the blood of individuals [19]. They are used as a biomarker for estimating the thymic output. DβTRECs develop during β-chain rearrangement and are generated before the proliferation phase [20]. The sj/Dβ T-cell rearrangement excision circles, known as the sj/Dβ-TREC ratio was studied in the pathogenesis of human immunodeficiency virus (HIV), and it was found that the sj/Dβ-TREC ratio was independently associated with HIV progression [21]. mTECs are unique stromal cells and play a significant role in central tolerance and autoimmunity through dealing with autoreactive cells via clonal deletion. Promiscuous gene expression, in which tissue-restricted antigens (TRAs) are expressed, is an important process in central tolerance and protection from autoimmunity. Peptides from TRAs are presented by mTEC to T-cells to undergo apoptosis or deviate them to be Treg cells that can inhibit self-reactive T-cells [22,23]. Insulin-like growth factor 2 (IGF2), which is synthesized by mTECs, is a dominant member of the insulin family. A direct proportional relationship has been found between tolerance to this peptide and its concentration in the thymus. The importance of thymus-dependent central tolerance has been reported; a defect in this central tolerance is considered to be the earliest process in the development of organ-specific autoimmune diseases [24,25,26,27].

## 3. Viral Infections and the Thymus Gland

**Figure 3:**
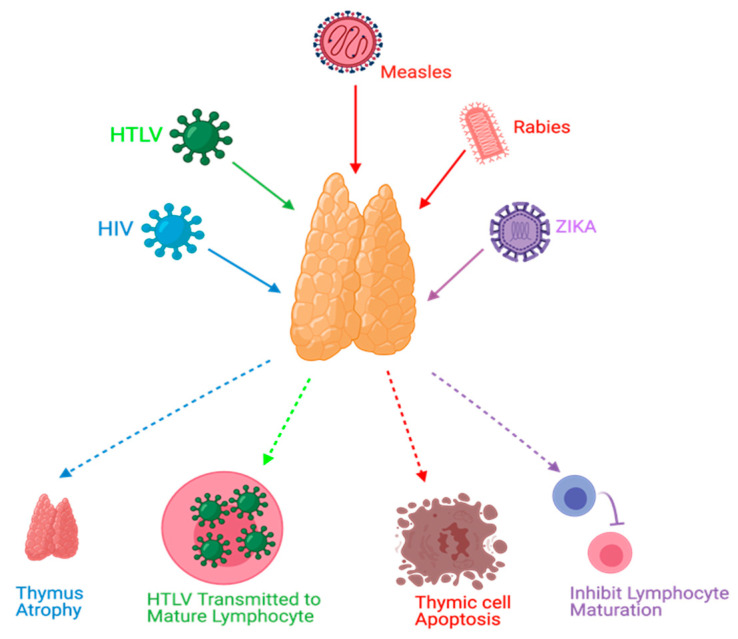
Effects of viral infections on the thymus. HIV causes thymus atrophy. HTLV transmission occurs through mature lymphocytes. Measles can cause thymic cell apoptosis. Rabies and Zika virus infections can inhibit lymphocyte maturation.

Viral infections can manipulate thymopoiesis and the thymus environment, possibly leading to phenotypic and functional modifications in the thymus. Different viruses have been reported to play a role in thymus dysfunction [21,28,29,30] (Figure 3).

Thymus functions during an HIV infection, which belongs to the retrovirus family, have been extensively studied. The mechanisms of these dysfunctions might include, but are not limited to, direct infection of the thymus by HIV, thymus structural changes, and apoptosis [21,31]. Moreover, it has been found that the thymus can retain its normal function following highly active antiretroviral therapy (HAART) in HIV-infected individuals, and this can be a useful predictor to ascertain HAART effectiveness [19,31,32]. Furthermore, thymic dysfunctions have been associated with HIV progression [21,28]. Human T-cell lymphotropic virus (HTLV), which belongs to the same family of HIV, can target the thymus. TECs play a role in the pathogenesis of HTLV-1 infections and act as a reservoir to transmit the virus to T-lymphocytes [33]. Rabies is a viral disease that has been found to target the thymus gland in animal models. Some reported mechanisms of rabies virus infection were apoptosis, depletion of thymocytes, and suppression of cell-mediated immunity [34,35,36,37,38]. Thymic apoptosis has also been reported in mouse hepatitis virus infection in animal models [39]. Measles was also found to cause thymic apoptosis in a murine model and terminal differentiation of TECs in human cortical thymic epithelial cell lines [40,41]. Moreover, Measles virus was detected in TECs of humans during the acute phase of infection and it can grow in the thymus of monkeys [40,42,43]. Ebola virus was also found to cause thymic infection in a murine model [44]. Cytomegalovirus (CMV) was studied in a murine model and found to replicate and persist in the thymic medulla, rather than the cortical region. Moreover, TECs were the primary hosts for viral replication. This infection was highly reduced when treated with antivirals that target CMV [45]. Zika virus was recently discovered to be able to infect the thymus, specifically TECs; this could affect thymocyte development [46]. TREC was used as a potential marker in respiratory syncytial virus (RSV) infections in which TRECs can be reduced during the severe presentation of RSV infections [47].

## 4. Coxsackievirus B4, the Thymus Gland, and Type 1 Diabetes

Environmental agents have been suggested as an important factor for clinical autoimmune disease development [48,49,50]. The genetic background alone cannot explain all T1D cases [51]. For example, the concordance index of T1D in monozygotic twins is less than 50%, and there is a huge variation in T1D incidence in different regions globally [4,52]. Infections, immunotherapy, endocrine disruptors and sex steroids, nutrition and vitamin deficiency, gut microbiota, and stress have been suggested as important environmental factors that may contribute to autoimmune diseases [48,49].

EVs can lead to T1D development via various mechanisms and through an interplay between the virus and the innate and adaptive immunity [10,53,54,55]. The relationship between T1D and EVs has been well established in human and animal models, and CVB4 persistence in monocytes/macrophages, gut mucosa, the pancreas, and thymus is suggested as a major mechanism in the pathogenesis of T1D [10,11,12,54,55,56]. Indeed, CVB4 can infect and persist in pancreatic cells, especially islet β-cells and ductal cells [57,58,59]. Moreover, the infection of monocytes/macrophages in vitro and in vivo by CVB4 has been reported by our team, and infection of these cells can lead to the production of IFN-α and inflammatory cytokines, which have a role in autoimmune response stimulation against β-cells [60,61,62,63].

In addition, CVB4 infection of monocytes/macrophages in vitro and in vivo has been reported to be enhanced by non-neutralizing anti-CVB4 IgG obtained from immune serum, which results in production of IFN-α and inflammatory cytokines [60,61,62,63,64,65]. This antibody-mediated enhancement of CVB4 infectivity increased viral load in organs resulting in a more severe outcome of the infection such as tissue lesions and hyperglycemia in mice challenged with CVB4 E2 [63,66,67,68]. Since T-cell repertoire alterations have been described in patients with T1D [69,70,71], it cannot be excluded that a CVB infection of the thymus could disturb central tolerance to insulin-secreting pancreatic β-cells and could then play a role in the pathogenesis of T1D.

Our team investigated the effects of in vitro and in vivo CVB4 infection on the thymus and thymic cells. Thus, it was reported that CVB4 can infect and persist in primary cultures of human TECs, leading to higher levels of cytokines interleukin-6 (IL-6), leukemia inhibitory factor (LIF), and granulocyte-macrophage colony-stimulating factor (GM-CSF) in their supernatants than in those of controls [72]. Moreover, it was found that CVB4 can infect human fetal thymus organ cultures (FTOC) leading to upregulation of MHC class I molecules and severe depletion of CD4+CD8+ thymocytes [73]. CVB4 infection of murine FTOC also disturbs the T-cell maturation and differentiation process [74]. The infection of the thymus of mice exposed to CVB4 has been investigated. CVB4 was orally inoculated in outbred Swiss mice, and the authors found viral RNA in the thymus, spleen, and blood up to three months post-inoculation [75]. Furthermore, the maternal–fetal transmission of CVB has also been described in the development of diseases affecting fetuses, newborns, and young infants [76,77,78]. Thus, the vertical transmission of CVB4 in mice and its possible association with T1D pathogenesis were investigated. CVB4 has been reported to infect the murine fetal thymus in utero and disrupt the homeostasis of thymic T-cell subpopulations that may play a role in the autoimmune development process [79,80,81]. We recently reported that the in utero CVB4 infection results in a significant decrease in sj and DβTREC in both the thymus and spleen of fetus and newborns together with a decrease in intra-thymic protein tyrosine kinase 7 (PTK7) expression (identified as a surface marker for recent thymic emigrants) and T-cell accumulation within the thymus [20]. These observations suggest a disturbance in the export of T-cells to the periphery that may interfere with T-cell maturation and several anomalies in thymic T-cell subsets were observed in the thymus from fetus of mice infected with CVB4 [12,20,79]. However, the infection of thymus or thymic cells with other CVB serotypes has not been studied. Whether EV infection of the thymus could interfere with the establishment of central tolerance to insulin-secreting pancreatic β-cells has been addressed.

TECs exert an important role in tolerance induction. Promiscuous gene expression allows presentation, via TECs of numerous autoantigens (e.g., insulin), to newly developed T-cells during their selection. TECs express not only insulin but also the growth hormone IGF2. IGF2, which shares a high homology with insulin, is more readily found in TECs than insulin [82]. The emerging role of IGF2 in insulin tolerance has been shown, notably in mice lacking IGF2 that have less insulin tolerance [83]. Moreover, the IGF2 epitope (competes with the insulin epitope for the same MHC molecule, and IGF2 itself) was shown to exert immunoregulatory functions on B- and T-cells [84,85]. The effect of a persistent infection of TECs with CVB4 on genes of the insulin family was investigated using a murine TEC line of medullar origin (Murine thymic epithelial cell line MTE4-14 derived from C3H/J (*H-2^k^*) thymic neonatal lobes), characterized by the expression of IGF2 and IGF-1, but not insulin. Notably, a dramatic decrease in *Igf2* transcription and IGF2 production in long-term cultures was recorded in TEC cells persistently infected with CVB4, while IGF-1 transcripts were less affected [86]. Thus, IGF2, the self-antigen of the insulin family, has an important role in central tolerance of the insulin family [87]. The impaired expression of IGF2 by TECs might lead to a disturbance in the negative selection of self-reactive thymocytes and a decrease in Treg cell generation. These mechanisms can play a role in the autoimmune attack against β-cells [12,53,88].

The effect of an acute infection of TEC cells (MTE4-14 cells line) by CVB4 on IGF2 transcripts and upstream signaling events impacting IGF2 regulation was recently reported by our team. The infection with CVB4 resulted in a decrease in major IGF2 transcript V3 and minor IGF2 transcript V1, along with the decrease in pro-IGF2 (mature IGF2 was not detected in this cell line). Furthermore, the infection with CVB4 resulted in a decreased activity in two regions of the core promoter of IGF2 transcript V3. Though picornaviruses replicate exclusively in the cytoplasm of infected cells, in MTE4-14 cells, CVB4 can affect the IGF2 host cell transcription. Transcription factors can be cleaved or not transported to the nuclei, resulting in impaired transcription. The transcription factor STAT3 is not only essential for TEC development and survival but also for the induction of antigen-specific T-cell tolerance. This transcription factor regulates the promoter and mRNA expression of IGF2 in humans and mice. Interestingly, the phosphorylation of STAT3 decreased in MTE4-14 cells infected with CVB4 [89]. These observations suggest that the loss of STAT3 phosphorylation in TECs could result in a decrease in IGF2 P3 promoter activity, and consequently, in the decrease of IGF2 expression. Further explorations are needed to confirm the hypothesis of the connection between the loss of STAT3 phosphorylation together with the loss of IGF2 P3 promoter activity.

**Figure 4:**
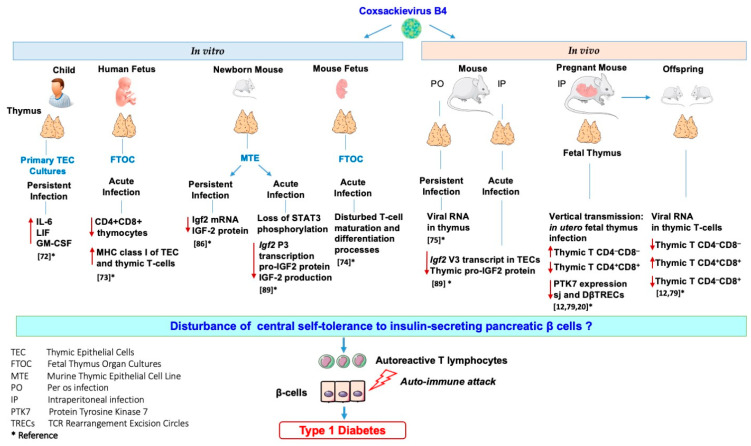
Effects of in vitro and in vivo Coxsackievirus B4 infection on the thymus and thymic cells: possible role in the disturbance of central tolerance to pancreatic β-cells.

CVB4 can infect mice following intraperitoneal or oral inoculation of the virus, as it has been previously reported by our team and others [67,68,75]. In TECs obtained from outbred mice infected intraperitoneally with CVB4, the dominant IGF2 transcript V3 and thymic IGF2 protein level had decreased [89]. Thus, CVB4 infection can modulate the expression of IGF2 in a model of TECs in vitro and can downregulate the IGF2 *V3* expression and protein synthesis *in vivo*. Therefore, infections with CVB4 can modulate the expression of thymic IGF2, which strengthens the hypothesis of a possible role of CVB infections in decreasing central tolerance to insulin. The observations concerning the effects of CVB4 infections on the thymus and thymic cells are summarized in Figure 4.

**Figure 5:**
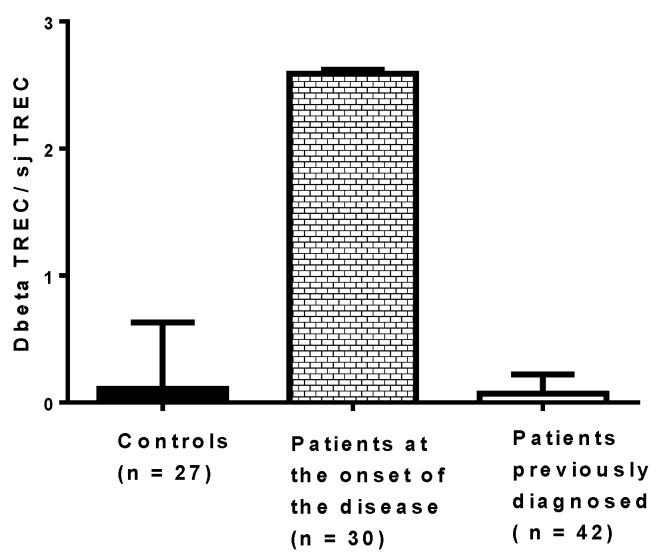
Ratio of DβTREC/sjTREC in newly diagnosed patients, previously diagnosed patients and controls. Type 1 diabetes was diagnosed a few days before collecting blood in patients at the onset of the disease, whereas the disease was diagnosed at least 10 months before collecting blood in the other group of patients.

It would be useful to assess the effect of virus infections on thymic functions in patients at risk of developing T1D, but it is challenging. The process of thymocyte maturation and education in vivo can be assessed through the quantification of two types of TRECs in peripheral blood mononuclear cells (PBMCs): DβTRECs formed during β-chain rearrangement and sjTRECs formed by the excision of the δ locus located inside the α-chain locus. The level of sjTREC and DβTREC in PBMCs of patients with T1D has been recently determined using qPCR, and the ratio of DβTREC/sjTREC, reflecting the thymus function, has been calculated (Figure 5). The ratio of DβTREC/sjTREC was significantly higher in newly diagnosed patients (*n* = 30) than in previously diagnosed patients (*n* = 42) (*p* < 0.01) and controls (*p* < 0.01), suggesting that the thymus function is disrupted in newly diagnosed patients (unpublished data). This observation supports the idea that the TREC level in PBMCs of patients with T1D can be useful to investigate the pathogenesis of the disease. Whether an impaired function of the thymus exists, as reflected by the ratio of DβTREC/sjTREC in prediabetic patients and in individuals with autoantibodies, warrants further research. Longitudinal studies are needed to determine whether enteroviral infections, especially CVB infections, result in a disturbance of thymic function that can be evaluated through the level of TRECs in PBMCs of individuals who developed the disease.

## 5. Conclusions

Dysfunction of the thymus gland is a crucial step toward the development of organ-specific autoimmune diseases such as T1D. Viruses can disrupt thymic functions through inducing conditions such as thymic atrophy, TEC dysfunction, apoptosis, and lymphocyte maturation impairment. EVs have been extensively linked to T1D pathogenesis, and this can be explained by EV-induced thymic dysfunction in the natal or perinatal life. Thymic infection by EVs can interfere with T-cell maturation or lead to autoreactive T-cell production, and both effects are potentially involved in T1D development. The results of experimental studies show that CVB4 infection can have multiple effects on the thymus (Figure 4). Whether these effects result in the dysregulation of tolerance involved in the pathogenesis of T1D is an issue that deserves further studies.
